# Stage-related outcome for thymic epithelial tumours

**DOI:** 10.1186/s12893-018-0434-z

**Published:** 2019-04-24

**Authors:** Valentina Tassi, Jacopo Vannucci, Silvia Ceccarelli, Alessio Gili, Alberto Matricardi, Nicola Avenia, Francesco Puma

**Affiliations:** 1 Division of Thoracic Surgery, Department of Surgical and Biomedical Sciences, S. Maria della Misericordia Hospital, University of Perugia Medical School, Perugia, Italy; 2 Public Health Section, Department of Experimental Medicine, University of Perugia, Perugia, Italy; 3 General and Specialized Surgery, “Santa Maria” Hospital, Department of Surgical and Biomedical Sciences, University of Perugia Medical School, Terni, Italy

**Keywords:** Thymic epithelial tumours, Thymoma, Thymic carcinoma, Surgery, Masaoka staging system, Oncological outcome, Iterative surgery

## Abstract

**Background:**

Thymic epithelial tumours (TETs) are characterized by a wide variety of biological behaviors. Radical resection and stage are strong prognostic factors. Aim of this study is to review our Single Center Experience.

**Methods:**

One hundred and seventy-seven patients observed in the period from January 2000 to December 2016 were included in the study. Data regarding clinicopathologic features, treatment, and survival were collected. Stage-related clinical standpoints and therapeutic options were also evaluated.

**Results:**

Non-surgical treatment was primarily performed in 15 (8.47%), unresectable disease was intraoperatively found in 12 cases (7.4%). The analysis of 150 patients undergoing curative surgery revealed 70 stage I TET (46.66%), 49 stage II (32.66%), 19 stage III (12.66%), 6 stage IVa (4%) and 6 stage IVb (4%) at the first hospital admission. Histology identified 12 A thymoma (8%), 38 AB (25.33%), 24 B1 (16%), 50 B2 (33.33%), 19 B3 (12.66%) and 7 carcinomas (4.66%). The mean follow up time was 84.14 months (sd = 61.68 months). Disease relapse occurred in 13 patients (8.78%) at a mean period of 78.85 months (sd = 60.87 months) after surgery. Exitus due to thymoma happened in 6 cases (4.05%) after a mean survival of 56.02 months (sd = 25.17 months). The 5-year overall survival rate was 0.94 (95%CI 0.88–0.97) and the 5-year disease-free survival rate was 0.90 (95%CI 0.83–0.94). The 5-year overall survival rates were 96.1% (95% CI, 89.9–98.5%) for the early stages and 87.4% (95% CI, 65.6–95.8%) for the advanced stages (*p* = 0.670). The 5-year disease-free survival rates resulted being 98.8% (95% CI, 92.3–99.8%) for the early stages and 59.8% (95% CI, 37.8–76.2%) for the advanced stages (*p* < 0.001).

**Conclusions:**

Advanced stage TETs are characterized by higher mortality and recurrence rates. Although technically demanding, surgery, as part of multimodality therapy, could prolong survival. Iterative surgical treatment of recurrences is a viable option for selected patients.

**Trial registration:**

The study was approved by the Institutional Review Board of Perugia and Terni University Hospitals [Code T1003] and was retrospectively registered.

## Background

Thymic epithelial tumours (TETs) are the most common epithelial neoplasms of the anterior mediastinum whose natural history proves a wide variety of biological behaviors [1–3]. Even if they are usually characterized by an indolent course, in some cases TETs can show an aggressive disease trend. [4–6]. The large number of scientific contributions did not thoroughly lead to complete agreement so far; this circumstance is mainly due to the retrospective study designs and the relatively low incidence of these particular tumours [7].

We reviewed our experience with TETs, in order to highlight biological and clinical aspects possibly concerning survival, disease progression and treatment.

## Methods

The experience with thymoma and thymic carcinoma of the Thoracic Surgery Unit of Perugia and Terni, in the period from January 2000 to December 2016, is retrospectively reviewed. Every patient’s workup included computed tomography scanning (CT), electromyogram (EMG), anti-acetylcholine receptor antibodies (ARAb) assay, complete blood count. Bronchoscopy and 18-fluorodeoxiglucose positron emission tomography (18-FDG PET/CT) were performed in particular cases. Magnetic Resonance Imaging (MRI) was occasionally indicated. Suspect diagnosis was usually achieved by imaging and clinical features but 56 cases (31.63%) with non-evocative clinical patterns have been submitted to biopsy as a first invasive procedure. Diagnostic procedures to assess clinical staging, tumour histology and myasthenia gravis occurrence in patients with clinically suspected thymic epithelial tumours are listed in Table 1.

**Table 1:** Diagnostic procedures to assess clinical staging, tumour histology and myasthenia gravis occurrence in patients with clinically suspected thymic epithelial tumours

Clinical staging and resectability	Chest X-rays
	CT scan
	MR imaging
	18FDG-PET/CT
	Bronchoscopy
Myasthenia Gravis	Neurological examination (medical history, physical examination)
	Anti-acetylcholine receptor antibodies assay
	Electromyogram (EMG)
Histology	Fine needle agobiopsy
	Surgical biopsy

All inpatient records were evaluated as well as the follow up data based on hospital registers, referring physician’s reports and family doctor’s databases. All patients have been then examined and asked about general condition, tumor-related symptoms and perceived quality of life.

Demographic facts, comorbidities, Myasthenia Gravis (MG) occurence and medical history were annotated. Histology (WHO classification) [8], Masaoka stage [9] and treatment (surgery, chemotherapy, radiotherapy, multimodality) were listed. Finally, disease-free survival (DFS), overall survival (OS) and therapeutic strategies for recurrences were entered into the analysis.

Patients were stratified in subgroups according to the stage (Masaoka I, II, III, IVa and IVb) in order to specifically emphasize stage-related clinical standpoints and the more appropriate therapeutic options.

### Statistical analysis

The data are expressed as mean and standard deviation (sd) unless otherwise stated. Survival and time to recurrence were calculated since the day of the primary treatment. Mean follow-up time was calculated using the reverse Kaplan-Meier method. Overall survival and disease-free survival were estimated by the Kaplan-Meier method. Survival curves were compared using the log-rank test. All estimates were achieved using STATA 14.2 (Stata Corp Ltd., College Station, TX, USA).

## Results

The series includes 177 consecutive patients, 91 males (51.41%) and 86 females (48.58%); the mean age was 63 years (sd = 13 years). Non-surgical treatment was primarily performed in 15 (8.47%) (10 unresectable stage III, 3 stage IV); two patients refused to undergo any surgical treatment after biopsy proven A thymoma. One hundred and forty-nine (84.18%) underwent primary surgical resection while post-chemotherapy resection was carried out in 13 patients (7.34%). Unresectable disease was intraoperatively found in 12 cases (7.4%). Adjuvant radiotherapy was administered in 34 cases (22.66%) (3 stage I, 11 stage II, 15 stage III, 5 stage IVa disease) whereas adjuvant chemotherapy was indicated in 10 cases (6.66%) (one stage I, one stage II, 2 stage III, 2 stage IVa and 4 stage IVb disease).

The analysis of patients undergoing surgery, revealed 70 stage I TETs (46.66%), 49 stage II (32.66%), 19 stage III (12.66%), 6 stage IVa (4%) and 6 stage IVb (4%) at the first hospital admission.

Histology identified 12 A thymoma (8%), 38 AB (25.33%), 24 B1 (16%), 50 B2 (33.33%), 19 B3 (12.66%) and 7 carcinomas (4.66%). MG occurred in 42 cases (28%) (recorded at diagnosis) and 15 (10%) were subclinical (positive instrumental tests but no symptoms complained). We recorded 5/150 myasthenic crises (3.33%) in the early post-operative course whereas we had 10/108 (9.25%) events of newborn myasthenia gravis in previously asymptomatic patients during follow up.

Two patients were lost during follow up. The mean follow up time was 84.14 months (sd = 61.68 months). Disease relapse occurred in 13 patients (8.78%) at a mean period of 78.85 months (sd = 60.87 months) after surgery.

**Fig. 1:**
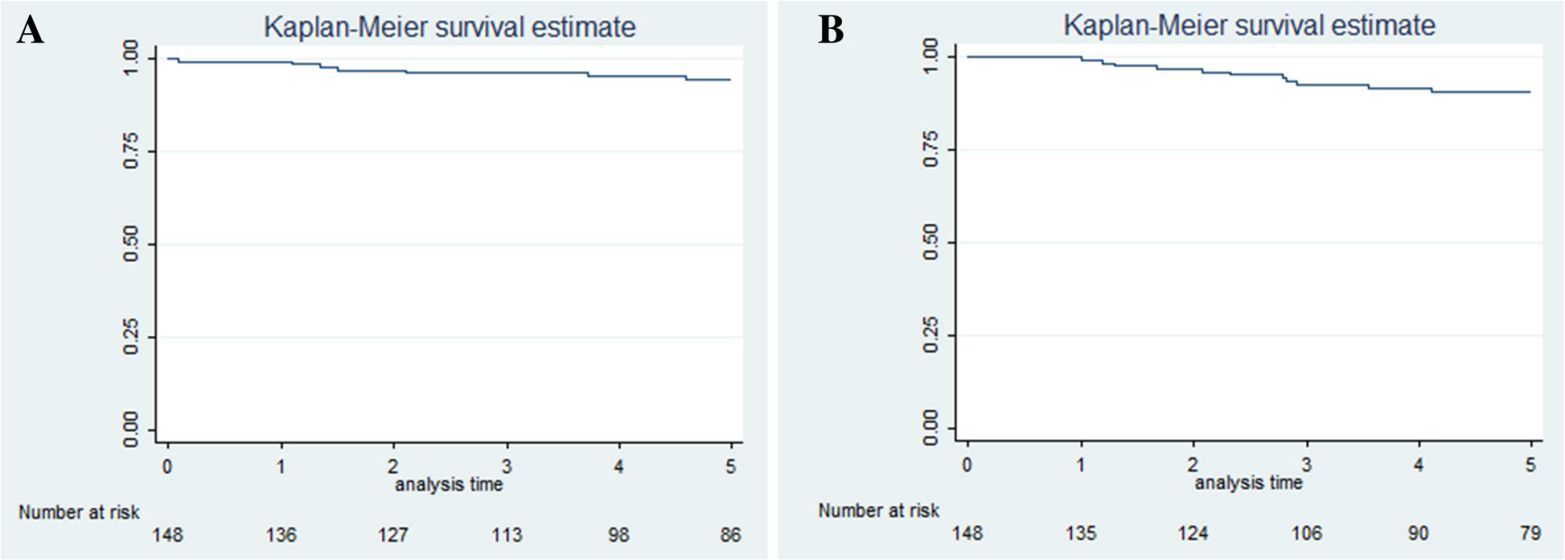
Kaplan-Meier survival curves for 148 patients surgically treated for TET (2 patients were lost at follow-up). Overall survival (**a**) and disease free survival (**b**) curves for the study population

**Fig. 2:**
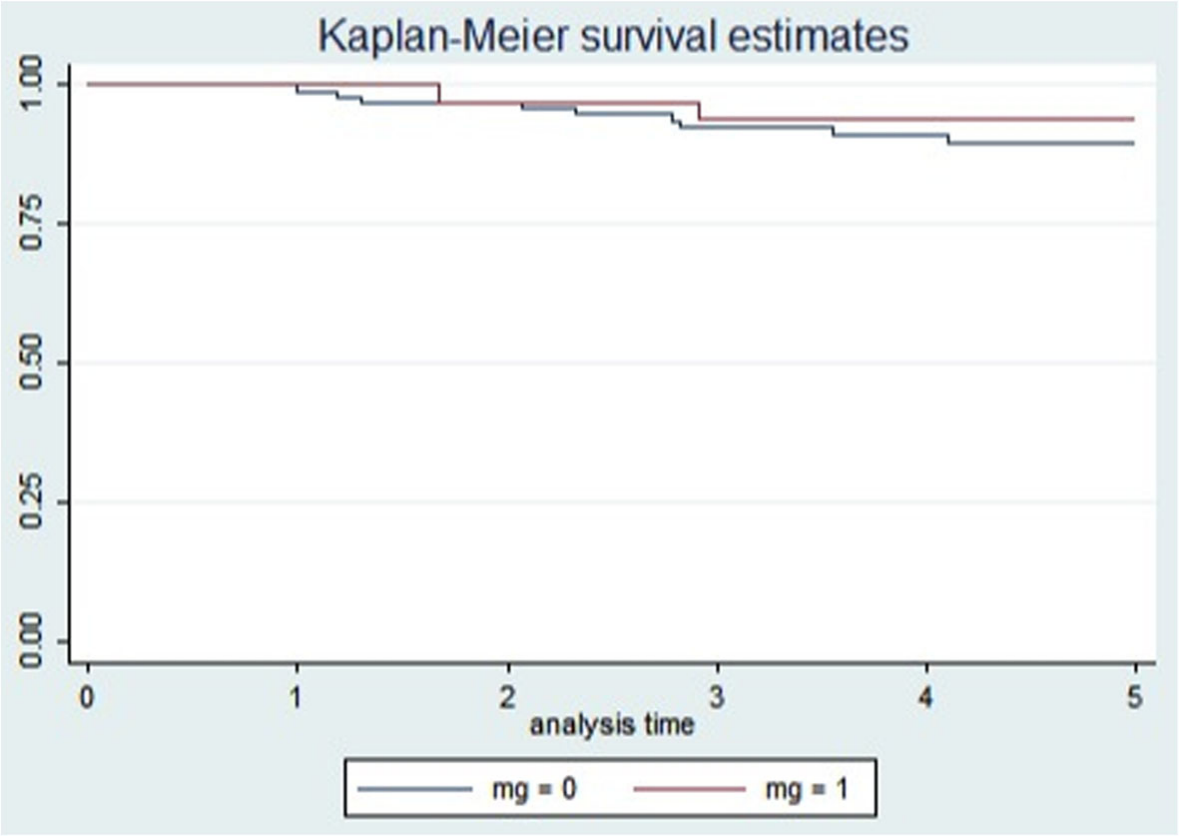
Kaplan-Meier survival curves for non-myasthenic and myastenic TET patients. Disease free survival curves for non-myasthenic (blue line) and myasthenic (red line) patients

Exitus due to thymoma occurred in 6 cases (4.05%) (4 stage III and 2 stage IVB, 1 B3, 2 B2, 3 carcinomas) after a mean survival of 56.02 months (sd = 25.17 months). The 5-year overall survival rate was 94.3% (95%CI 88.3–97.2%) and the 5-year disease free survival rate was 90.8% (95%CI 83.9–94.8%). Figure 1 shows the overall survival (A) and disease-free survival (B) curves of the study population. The 5-year disease free survival rate resulted being 89.6% (95%CI 80.9–94.5%) for non myasthenic patients and 93.7% (95%CI 77.1–98.4%) for myasthenic (*p* = 0.501). Figure 2 shows the disease-free survival curves for non-myasthenic and myasthenic patients.

### Stage-related histology and treatment

Stage I (70): 4 A thymoma (5.71%), 25 AB (35.71%), 12 B1 (17.14%), 26 B2 (37.14%), 3 B3 (4.28%). Neoadjuvant therapy was administered to one patient (1.42%) with a 20 cm-thymoma. Sixty patients underwent total thymectomy and 10 thymomectomy through median sternotomy (60), cervicotomy (1), thoracotomy (6) and video-assisted thoracic surgery (VATS) (3). For the resection of the aforementioned giant thymoma, a femoro-femoral cardiopulmonary bypass was planned but not established. There was no R1 thymectomy and 2 patients (2.85%) received adjuvant radiotherapy. Recurrence happened in one case (1.42%) (pleural). MG was present in 17 at diagnosis (24.28%) and 6 had a late onset (8.57%).

Stage II (49): 7 A thymoma (14.28%), 12 AB (24.48%), 8 B1 (16.32%), 13 B2 (26.53%), 7 B3 (14.28%) and 2 carcinomas (4.08%). Neoadjuvant therapy was carried out in one thymic carcinoma patient (2.04%). Preoperative embolization of the feeding arteries was performed in 2 cases for highly vascularized giant thymomas. Eight patients underwent thymomectomy and 41 total thymectomy through median sternotomy (40), thoracotomy (4), VATS (3), Clamshell incision (1) and sterno-thoracotomy combined access (1). There was no R1 thymectomy and 10 patients (20.40%) received adjuvant treatment (9 radiation, one chemo-radiation). Recurrences did not happen in this subgroup. MG was present in 18 at diagnosis (36.73%) and 3 had a late onset (6.12%).

Stage III (19): one A thymoma (5.26%), 3 B1 (15.7%), 7 B2 (36.84%), 6 B3 (31.57%) and 2 carcinomas (10.52%). Induction chemotherapy was performed in 2 (10.52%) based on supposed non-resectable stage III at imaging. Surgical resection was performed through median sternotomy (17) and sterno-thoracotomy combined access (2). Total thymectomy was carried out en bloc with pleura or pericardium in 9 cases, phrenic nerve in 7, pulmonary resection in 10 (7 wedge, two segmentectomies, one lobectomy) and superior vena cava (SVC) system in 9 (one reconstruction with polytetrafluoroethylene (PTFE), one reconstruction with pericardial patch, 2 direct suture and 5 left brachiocephalic vein resection). The case of SVC resection and reconstruction with pericardial patch was performed under veno-venous right internal jugular vein- right atrium bypass. There was one mediastinal R1 (5.26%) after surgery and 18 patients (94.73%) received adjuvant treatment (2 chemotherapy, 16 radiotherapy). Recurrences happened in 5 cases (27.7%) (one mediastinal, 3 pulmonary and one nodal relapse). MG was present in 4 at diagnosis (21.05%) and one had a late onset (5.26%).

Stage IVa (6): one AB (16.66%), one B2 (16.66%), 3 B3 (50%) and one carcinoma (16.66%). Neoadjuvant therapy was administered to one patient (16.66%). The surgical access was sternotomy in 3 cases, thoracotomy in 2 and combined sterno-thoracotomy in one patient. The thymic tumor was resected en bloc with pleura or pericardium (4), phrenic nerve (1), pulmonary wedge resection (2). Multiple pleural and pericardial nodules were resected in all cases, but the complete pleurectomy was required in 3. There were 3 R1 (50%) (at pleural level) after the surgical procedure and 5 patients (83.33%) received adjuvant treatment (2 chemotherapy, 3 radiotherapy). All R1 patients experienced multiple new pleuro-pericardial implants and pulmonary metastasis. MG was present in 2 at diagnosis (33.33%).

Stage IVb (6): one B2 (16.66%), 3 B3 (50%) and 2 carcinomas (33.33%). Neoadjuvant therapy was administered to 2 patients (33.33%). The surgical access was sternotomy in 2 cases, thoracotomy in 4. TET was resected en bloc with pleura or pericardium (2), phrenic nerve (1), pulmonary lobectomy (1), left pneumonectomy (2). In four patients nodal metastases (2) and pulmonary metastasis (2) were resected. Partial pleurectomy and diaphragmatic resection were required in 2 cases. There were 2 R1 (33.33%) (at pleural level) after the surgical procedure and 4 patients (66.66%) received adjuvant treatment (chemotherapy). Recurrences happened in 4 cases (66.66%) (2 pleuro-pulmonary and 2 nodal relapses). MG was present in one patient at diagnosis (16.66%).

### Recurrences

Recurrent TETs were 13: one in the primary site, 4 pleural-pericardial and 8 distant metastases (5 in the lung, 3 in the nodes). They appeared in one B1 thymoma, 6 B2 and 3 B3 and 3 carcinomas. Seven patients (53.84%) underwent one or more surgical procedures for the treatment of pleural, pericardial, pulmonary or nodal recurrences. Two of them (28.57%) died for TET progression, three (42.85%) are alive with stable disease and two (28.57%) are alive with no evidence of disease. Six patients (43.15%) received chemo-radiation therapy. Four of them (66.66%) died for TET progression and two (33.33%) are alive with stable disease.

### Stage-related follow up

**Fig. 3:**
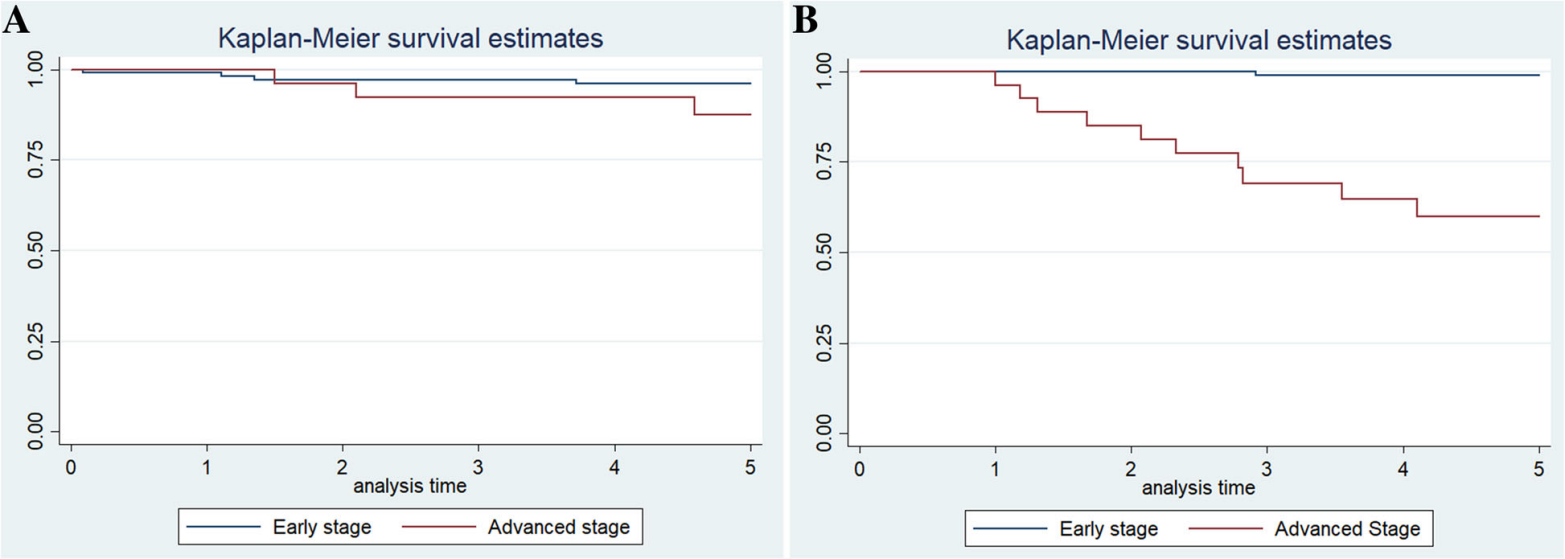
Kaplan-Meier survival curves for early and advanced stages of TET. Overall survival (**a**) and disease free survival (**b**) curves for early (blue line) and advanced (red line) stages

The 5-year overall survival rates resulted being 96.1% (95% CI, 89.9–98.5%) for the early stages and 87.4% (95% CI, 65.6–95.8%) for the advanced stages (*p* = 0.670). The 5-year disease-free survival rates resulted being 98.8% (95% CI, 92.3–99.8%) for the early stages and 59.8% (95% CI, 37.8–76.2%) for the advanced stages (*p* < 0.001). Figure 3 shows the overall survival (A) and disease-free survival (B) curves for early (I and II) and advanced (III and IV) stages of TET.

### Thymoma in cysts

Thymoma in cysts (2 cases) could be a tricky situation. In some cases the diagnosis of thymoma is unexpected after simple thymic cyst removal (no cases in our series). Surgery has to face a really thin cystic wall that needs to be respected. Thymoma in cysts is really rare and cyst-related risk of malignancy is not assessable out of our experience.

## Discussion

### Stage I and II

This is the least aggressive pattern that TETs can show: mainly asymptomatic often diagnosed by chance [1, 10]. Surgery (thymus gland removal) is the standpoint therapy and proved to be effective and safe [11, 12]. If the resection is radical, the complete tumor excision leads to a bright prognosis [10, 11]. In Masaoka stage I and II thymoma, surgery is actually a potential method of cure by itself: adjuvant radiotherapy is not of evident help following appropriate surgical resection [1, 10]. Sternotomy approach remains the most common technique to be employed but, along with thoracotomy, there are several series describing the minimally invasive surgery as a valuable therapeutic option in the removal of a mediastinal mass suggesting a low-stage thymoma [13–15]. The recurrence seems to be frequently related to spillage, malignant residue after suboptimal surgery or possible multifocal malignancy within the mediastinal fat [1, 5, 12, 16]. A careful surgery can safely remove all the neoplastic tissue but must be respectful of the primary lesion integrity [17]. Open surgery represents the standard approach; however, in the last decades, minimally invasive techniques have gained popularity [1, 2]. Encouraging results have been reported in large series from high volume centers [13, 15, 17]. Tumor manipulation during minimally invasive procedures can result in additional risk of drop metastasis that would forward the stage from I-II to IVa with a significant worsening of prognosis [16–18]. Therefore, the indication to such operations needs to be balanced against its hazards [15, 17, 18]. Moreover, the oncological potentials of the robot-enhanced thoracoscopic resections have to be validated by long-term trials [13, 19].

Stage I and stage II lesions cannot be separately discussed [1, 11]. Preoperative imaging plays an important role not only to distinguish early from advanced stages but it can also be useful to plan the optimal surgical approach for TET [20, 21].

Finally, even if a small, round well-incapsulated lesion can hopefully depict a benign tumor, TETs have always to be considered malignant due to their potentials and natural history [3, 6].

### Stage III

The tumor infiltration of a proximal organ varies largely from minimal lung involvement to extended compromise of great vessels [2, 12, 20]. The spectrum of possible therapy is dependent on the tumor resectability [22]. Preoperative imaging plays an important role, but the sensibility of the commonly employed technologies (CT, MRI and PET-CT) does not guarantee a complete tumor removal earlier than the time of surgery [20, 22]. Although sternotomy is the ordinary approach, it is not the only access available: thoracotomy and other incisions have been successfully employed, but no kind of minimally invasive surgery has a reasonable indication [17]. The difficult preoperative assessment for complete resectability, probably represents the primary pitfall and concurs in producing the substantial rate of subtotal removal [21, 23]. This fact underlines that the surgical accuracy is the unique non-biological key point to deal with this disease [1, 11, 12]. Although the complete resection is the aim to achieve, it remains a macroscopic presumption after any kind of surgery. Considering the potential microscopic spread and the following relapse, minimal tumor remnants after surgery for a stage III thymoma have been reported in different series [24, 25]. For this reason, adjuvant treatment can appropriately be indicated [1, 2, 24].

Preoperative treatment can be performed in order to down-stage the disease and make the resection feasible [22]. Whereas radiotherapy has a valuable post-operative recommendation, neoadjuvant treatment is mainly based on chemotherapy, with multiple therapeutic schemes that can be employed with satisfying results [22, 26]. We agree with those who support the theory that, among stage III, there are several different conditions regarding the compromised organs by direct infiltration from the tumour [1, 2, 27]. According to the new TNM/IASLC staging system, indeed, stage III tumours have been divided in stage IIIa and stage IIIb tumours, in order to better identify patients suitable for surgery. Heart and great vessels, as long as trachea and esophagus involvement, should be considered a more advanced tumor compared to those cases with lung, pericardium, chest wall, phrenic nerve or SVC infiltration [1, 27].

### Stage IV

Surgery is part of a multimodality treatment and timing for surgery should be indicated in a dedicated multidisciplinary setting where inappropriate therapeutic decisions would be minimized [1, 2].

Surgery can manage both primary lesions spreading to pleural space or recurrent metastatic pleuro-pericardial seedings [28]. The aim of surgery does not change at all: debulking or cytoreduction have minimal rationale; surgery has a role in case of possible complete removal [24]. Once decided to submit the patient to surgery for stage IV thymoma, diagnostic and staging information must satisfy technical conditions for a possible macroscopically complete resection; if it is absolutely not achievable, any surgical plan should be abandoned [25, 28]. Redo-surgery is not self-limiting and cases of indolent disease can develop multiple metachronous metastasis that can undergo surgery time by time [16, 29].

Surgery is represented by a large number of procedures that can be advocated [24, 28]. The entire removal of each spot of malignancy can rise from minimal lung resections for visceral pleura seedings to wide pleurectomy and pleuropnemonectomy for extensive disease [1, 24].

Extended resections for stage III and IV thymoma can be technically demanding and the high morbidity and possible mortality must be taken into account [24, 28]. The balance between risk and effectiveness is indeed crucial [24, 25].

### Off stage

There are possible situations in which Masaoka staging, histology and ordinary preoperative evaluations are of limited importance. Life-threatening conditions are really rare; however, in these circumstances such as spontaneous rupture, bleeding and possible other emergency conditions, the treatment can be surgical, but sometimes impossibly radical, or decided at the time of surgery for insufficient diagnostic work up [30]. In case of ectopic thymoma or orthotopic tumor grown outside the mediastinum, the Masaoka stage can be even misleading; the principles of surgery for thymoma find in the complete removal of the thymus gland and the surrounding fat the basic procedure to be extended to the neighboring organs in case of infiltration [2]. For ectopic thymoma or orthotopic tumor grown outside the mediastinum, the treatment can be accomplished without total thymectomy [31]. We suggest to look for R0 instead of making a total thymectomy if the tumor is far detached from the thymus gland and to follow the patient up for a supposed enhanced risk of new primary thymic malignancies [31, 32].

### Thymoma and myasthenia gravis

Our series suggests that MG incidence is not associated to stage. Myasthenic patients presented a trend to a higher 5 year DFS compared to non-myasthenic ones, even if this parameter does not reach a statistical significance. Probably, this trend can be explained considering that the presence of the syndrome can lead to the diagnosis of an otherwise asymptomatic early-stage thymoma [1, 2].

## Conclusions

Advanced stage TETs are characterized by higher mortality and recurrence rates. Although technically demanding, surgery, as part of multimodality therapy, could prolong survival. Iterative surgical treatment of recurrences is a viable option for selected patients.

## Abbreviations

18-FDG PET: 18-fluorodeoxyglucose positron emission tomography
CI: Interval of confidence
CT: Computed tomography
DFS: Disease-free survival
MG: Myasthenia gravis
MRI: Magnetic resonance imaging
OS: Overall survival
PTFE: Polytetrafluoroethylene
sd: Standard deviation
SVC: Superior vena cava
TET: Thymic epithelial tumour
VATS: Video-assisted thoracoscopic surgery
WHO: World Health Organization
